# Why Contextual Preference Reversals Maximize Expected Value

**DOI:** 10.1037/a0039996

**Published:** 2016-07

**Authors:** Andrew Howes, Paul A. Warren, George Farmer, Wael El-Deredy, Richard L. Lewis

**Affiliations:** 1 School of Computer Science, University of Birmingham; 2 School of Psychological Sciences, University of Manchester; 3 Departments of Psychology and Linguistics and Weinberg Institute for Cognitive Science, University of Michigan

**Keywords:** expected value maximization, preference reversals, rationality, choice

## Abstract

Contextual preference reversals occur when a preference for one option over another is reversed by the addition of further options. It has been argued that the occurrence of preference reversals in human behavior shows that people violate the axioms of rational choice and that people are not, therefore, expected value maximizers. In contrast, we demonstrate that if a person is only able to make noisy calculations of expected value and noisy observations of the ordinal relations among option features, then the expected value maximizing choice is influenced by the addition of new options and does give rise to apparent preference reversals. We explore the implications of expected value maximizing choice, conditioned on noisy observations, for a range of contextual preference reversal types—including attraction, compromise, similarity, and phantom effects. These preference reversal types have played a key role in the development of models of human choice. We conclude that experiments demonstrating contextual preference reversals are not evidence for irrationality. They are, however, a consequence of expected value maximization given noisy observations.

One of the successes of the rational analysis of human cognition has been that a number of apparent irrational behaviors have been shown to be rational given different assumptions about what shapes adaptation (Hahn & Warren, 2009; Le Mens & Denrell, 2011; Oaksford & Chater, 1994; Trimmer, 2013). For example, Hahn and Warren (2009) have shown that a consideration of an individual’s experience of a fair coin toss and of the bounds imposed by working memory can explain seemingly biased perceptions of randomness. Similarly, Oaksford and Chater (1994) proposed that human reasoning might be understood as rational relative to the ecology of an uncertain world, rather than irrational relative to deductive logic. Despite such successes, one apparent irrationality of human behavior that continues to challenge the rational perspective is the *contextual preference reversal* (Huber, Payne, & Puto, 1982; Huber & Puto, 1983; Pettibone & Wedell, 2007; Soltani, Martino, & Camerer, 2012; Trueblood, 2012; Trueblood, Brown, Heathcote, & Busemeyer, 2013; Tversky & Simonson, 1993; Wedell, 1991). In the current article, we report an analysis of contextual preference reversals that shows that human choice can be considered computationally rational (Lewis, Howes, & Singh, 2014) given the uncertainty introduced by perceptual and cognitive capacities. The main contribution of this analysis is to show that preference reversals are inevitable signatures of a rational response to the structure of the decision task and simple assumptions concerning perceptual and cognitive processing. In particular, preference reversals are a rational consequence of noisy observations of subjective expected utility and of the ordinal relations between features. Despite the minimal nature of these assumptions, we show in this article that the theory predicts and provides deep explanations of several types of empirically observed preference reversals—empirical regularities that have played a key role in the development of process theories of choice that are not grounded in utility maximization.

Contextual preference reversals occur when a preference for one option (expressed through behavioral choice) over another is reversed by the availability of further options. Consider a simple example. When asked whether you would like a healthy apple *A* or a cake *B* that has 30 g of sugar, you might choose the apple. However, when told that a second cake *D* is available (a *decoy*) that has 40 g of sugar, you might switch preference to *B*. A person who makes such a switch makes a *contextual preference reversal*. Typically, in preference reversal experiments options are described explicitly in terms of two attribute dimensions and not just one. For example, in the experiments reported by Huber et al. (1982) the dimensions included the price and quality of beer. In the Wedell (1991) experiments participants were offered options with numeric probability and value features. In both of these circumstances, a choice between, say, *A* and *B* is difficult because *A* might be higher than *B* on one dimension, but lower on the other.

In the decision making literature, a taxonomy of preference reversal types has been introduced that depends on the relative positioning of options *A*, *B*, and *D* in the multidimensional attribute-value space. One well-studied type is the *attraction* effect. A gamble version of a preference reversal task with an attraction effect decoy is illustrated in Figure 1. Option *B* has a higher value *v* than option *A* but a lower probability *p*. Option *B* dominates *D* but not *A*. Options *A* and *B* have approximately equal expected value. In the Figure, given the position of *B*, *A* appears in the top left, and *D* in the rectangle below *B*. The presence of *D* tends to increase the proportion of *B* choices. This is known as an attraction effect because the decoy “attracts” choices to the target, where the target is the dominating option.[Fig-anchor fig1]

In addition to multiattribute decision problems (Huber et al., 1982; Huber & Puto, 1983; Simonson, 1989; Simonson & Tversky, 1992) and gambles (Herne, 1999; Wedell, 1991), other paradigms have been used to study contextual reversals. One study used political candidates as stimuli (O’Curry & Pitts, 1995). More recently, reversals have been observed in choices between rectangles with different areas (Trueblood et al., 2013) and they have been the subject of analysis in economics (Loomes, 2005). The effect has also been shown in zoology (Latty & Beekman, 2011; Parrish, Evans, & Beran, 2015; Schuck-Paim, Pompilio, & Kacelnik, 2004), including in birds (Bateson, Healy, & Hurly, 2003; Shafir, Waite, & Smith, 2002), bees (Shafir et al., 2002), and ameboid organisms (Latty & Beekman, 2011). In humans it can be attenuated by increasing blood glucose levels (Masicampo & Baumeister, 2008), elaborating the descriptions of attributes (Ratneshwar, Shocker, & Stewart, 1987), and by increasing the difference in expected value between the two options (Farmer, 2014). It has also been observed in perceptual-motor decisions (Farmer, El-Deredy, Howes, & Warren, 2015).

The fact that people make contextual preference reversals has been taken as evidence against normative theories of human choice. In particular, many have suggested that preference reversal phenomena indicate that people do not make independent evaluations of each option (Ariely & Wallsten, 1995; Huber et al., 1982; Louie & Glimcher, 2012; Simonson, 1989; Simonson & Tversky, 1992; Summerfield & Tsetsos, 2012; Tsetsos, Usher, & Chater, 2010; Tversky & Simonson, 1993). Preference reversals suggest that the values of each option are influenced by additions to the set of available options, which constitutes a violation of the *Independence from Irrelevant Alternatives* (IIA) axiom required in many value maximizing models (e.g., Luce, 1959). As its name suggests, the axiom demands that a preference between two options is not changed by the addition of an option to the set of what is available.

There is a common view that contextual preference reversals are evidence that people violate value maximization. This belief has survived for at least 20 years. According to Tversky & Simonson (1993, p. 1179):

The standard theory of choice—based on value maximization—associates with each option a real value such that, given an offered set, the decision maker chooses the option with the highest value. Despite its simplicity and intuitive appeal, there is a growing body of data that is inconsistent with this theory.

In the same article, Tversky and Simonson describe why they believe that context effects are evidence against value maximization (p. 1188):

The analysis of context effects, in perception as well as in choice, provides numerous examples in which people err by complicating rather than by simplifying the task; they often perform unnecessary computations and attend to irrelevant aspects of the situation under study.

More recently, Usher, Elhalal, and McClelland (2008, p. 297) state, “. . . contextual reversal effects . . . demonstrate a limitation of rationality in choice preference.” Simonson (2014, p. 1) states,

. . . the belief in irrationality is now widely accepted among the general public. The most commonly used operationalization of irrationality among decision researchers has been based on violations of value maximization. Preferring a dominated option or expressing different preferences depending on the framing of options . . . demonstrate[s] . . . the absence of stable preferences and resulting irrational decisions.

The main response in psychology and behavioral economics to the apparent empirical failures of normative models of decision making has been to pursue process theories of the mechanisms that underlie human choice. This approach embraces the distinction that the normative models prescribe what decision making *ought* to be like, and the psychological mechanism or process theories describe *how it actually works* in humans. Many of these process theories have indeed provided successful empirical accounts, and we briefly review several of the key contributions below. Nevertheless, despite the number and diversity of such theories, they have not been deployed with the purpose of explaining preference reversals as a rational adaptation.

In contrast to the process theories, our aim in the work presented here is to pursue a rational account that explains phenomena as a consequence of expected value maximization and cognitive constraints (Hahn, 2014; Howes, Lewis, & Vera, 2009; Lewis et al., 2014). This approach is, therefore, one way to pursue an understanding of human cognition as computationally rational (Lewis et al., 2014). This approach has precedents in other areas of cognitive science. For example, in the account of aimed movement offered by Meyer, Abrams, Kornblum, Wright, and Smith (1988), the movement strategy optimizes a speed–accuracy trade-off given constraints on the available noisy neuromotor control signals. The authors explicitly frame their contribution as relevant to the “degree to which mental processes incorporate rational and normative rules.” Similarly, in the analysis by Maloney and Mamassian (2009) of target selection for aimed movement, the optimal aim point is derived using decision theory given the assumptions that motor performance is noisy and that people try to maximize an objective function in which there is a trade-off between the number of points awarded for hitting a target and avoiding a penalty region. The aim point chosen by a participant is a consequence of the particular individual’s bivariate Gaussian distribution of end points around each possible aim point and the objective function. The aim point predicted by decision theory is the aim point that maximizes expected utility, and there is no need to fit the parameters of the decision model to the outcome data.

A number of contributions to the broader decision making literature have argued that preference reversals can be rational (Bordley, 1992; McNamara, Trimmer, & Houston, 2014; Shenoy & Yu, 2013; Trimmer, 2013). For example, Trimmer (2013) shows that, in an evolutionary context, even options that are never chosen by an animal can be relevant to a decision. Bordley (1992) argues that individuals have prior expectations about the value of lotteries and if these are taken into account then preference reversals will follow.^1^ A more detailed discussion of these contributions is provided at the end of the current article.

In the following sections of the article:

1 We review existing models of preference reversals in humans.
2 We present a new model of choice between options. The model is of a task where a human is asked to choose between options each consisting of a set of features, for example a *probability* and a *value*. This task is used in the preference reversal paradigm reported by Wedell (1991). The model combines two *noisy* observations of each option. The first observation is based on noisy *calculation* of the model’s subjective expected utility. The second observation is a noisy observation of *ordinal relations*, which is a noisy encoding of the partial ordering of the magnitudes of the presented probabilities and values. The model chooses the expected value maximizing option given these observations.
  The assumption that there is noise in perceptual and cognitive processes, and perhaps uncertainty in preferences, is uncontroversial (Costello & Watts, 2014; Faisal, Selen, & Wolpert, 2008; Hilbert, 2012; Loomes, 2005; Maloney & Mamassian, 2009; Seymour & McClure, 2008; Warren, Graf, Champion, & Maloney, 2012). The use of ordinal observations is consistent with recent evidence that people make ordinal feature comparisons in service of decisions (Noguchi & Stewart, 2014).
3 We show that the model predicts preference reversals. We also show that it obtains higher expected value than a model that only makes use of noisy calculation.
4 We apply the model to a previously reported empirical study of preference reversals (Wedell, 1991). The model predicts preference reversals in three conditions where it is observed in humans and predicts its absence where it is not.
5 We show that the model predicts related contextual preference reversal phenomena, namely the *compromise* and *similarity* effects (Maylor & Roberts, 2007; Trueblood, 2012; Trueblood et al., 2013; Tversky, 1972; Tversky & Russo, 1969). The compromise and similarity decoy positions differ from the attraction decoy positions illustrated in Figure 1. The compromise decoy might, for example, have a higher probability than *A* and a lower value, so as to make *A* a compromise between the decoy and *B*. The compromise decoy causes the intermediate item to be selected more often than it would otherwise. The similarity decoy, as its name suggests, has the same expected value as the other options and a very similar probability and value to one of them. The similarity decoy causes the similar option to be selected *less* often.
6 We use the model to generate predictions for the *phantom decoy* effect. The phantom decoy is positioned so as to dominate one of the other options on at least one dimension, but is not available for choice. The predictions are supported by the results of Soltani et al. (2012) but not by those of Pettibone and Wedell (2000, 2007). We discuss the contradictions between these studies.
7 We use the model to generate predictions for the effect of *time pressure* (Pettibone, 2012) on preference reversals. As time pressure increases preference reversal effects tend to diminish.
8 We discuss the implications of our findings for process theories of cognition, and contrast our model to others that also consider the rationality of preference reversals (Bordley, 1992; McNamara et al., 2014; Shenoy & Yu, 2013; Trimmer, 2013).

## Background: Models of Preference Reversals

We focus in what follows on models of human preference reversals in consumer product choice and gamble experiments. Because it is the primary contextual preference reversal type, we first introduce the attraction decoy task in more detail. We subsequently introduce the related decoys (the compromise, similarity, phantom, and inferior but nondominated decoys) in a later section of the article.

**Figure 1 fig1:**
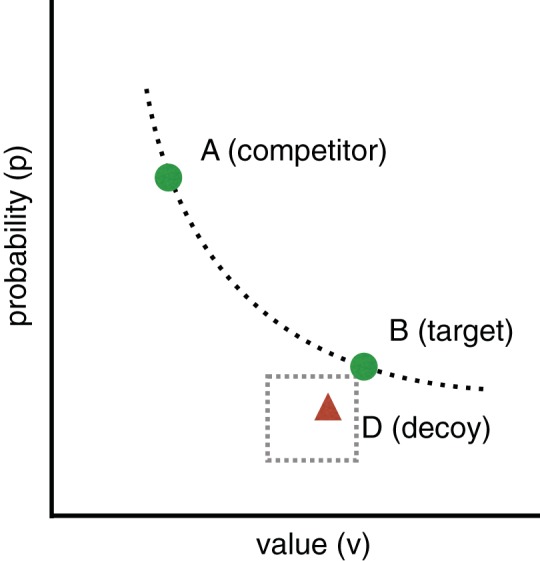
In one type of contextual preference reversal experiment two options *A* and *B* are each described in terms of two features, for example a probability *p* and value *v*. *A* has a higher probability and *B* has a higher value. *A* has the same expected value as *B* and they are, therefore, both on the same line of equal expected value (the dotted curve). A decoy *D* is placed in the rectangle to the left and below either *A* or *B* (*B* in the figure). The decoy in the figure is dominated by *B* but not by *A*. See the online article for the color version of this figure.

The attraction effect preference reversals, as illustrated in Figure 1, are observed experimentally when participants are asked to choose between options from various product categories including cars, restaurants, and beers (Huber et al., 1982). Each pair of options presented to participants shared two features such as price and quality. Two options *A* and *B*, with approximately equal utility such that each dominated the other on one of the features were presented. Two weeks after choosing between *A* and *B* in each of the categories, 93 participants returned to answer the same choice problems but with the decoy *D* added. The result, aggregated across product categories, was a nine point increase in percentage share for the option that dominated the new decoy. A follow up study (Huber & Puto, 1983) showed that the effect could also be achieved when the decoy is inferior to, but not strictly dominated by, one of the options.

The attraction effect has also been shown in choices between gambles (Wedell, 1991). The stimuli all had an expected value of approximately $10, but with different probabilities and values. For example, one problem was to choose between (.83, $12), (.67, $15), and (.78, $10). The last gamble in this set, the decoy, can be ruled out because both its *P* and its *V* are less than those in the first gamble. Choosing between the first two gambles is difficult because while one gamble dominates on *P*, the other dominates on *V*. A paired task was to choose between (.83, $12), (.67, $15), and (.62, $13). The first two gambles are the same as before but the new decoy is dominated by the second gamble rather than the first. In this paradigm, preference reversals can be measured as the proportion of pairs on which the dominating gamble is chosen in both variants. This study resulted in about 20% of choices exhibiting the attraction effect preference reversal.

There have been many attempts to explain preference reversals in terms of underlying psychological processes. According to one early account, Range-Frequency Theory (Parducci, 1974), option *D* in Figure 1 extends the range of values on the *y*-axis thereby making *B*’s loss to *A* appear smaller than if option *D* were not present (Huber et al., 1982). In motivational accounts, it has been proposed that a participant’s desire to be able to justify their choice to the experimenter leads them to prefer a dominating option (Simonson, 1989). The argument is that it is easier to justify choosing *B* because it dominates one of the other options and *A* does not.

Tversky and Simonson (1993) proposed that preference reversals can be explained in terms of two psychological processes: a process that weights the effect of the background to the decision, and a comparison process that describes the effect of the local context. The local context might consist of a choice between three beers, and the background, experienced before the local context, might have included five or six other beers. The background process increases the value of options in the local context if, for example, they have a price that is lower than prices of beers in the background. The local context process increases the value of options that are better than proximal options that are also in the local context, and it does so by summing the relative advantage that each option has over other options in the set (Tversky & Simonson, 1993, p. 1186). The model calculates the relative advantage that each option has over other options on each feature. In the attraction effect, the dominating option’s relative advantage over the decoy exceeds the relative advantage of the other option over the decoy. Therefore, when the nondecoy options are compared only with each other, they will have equal choice probability, but when the decoy is included in close proximity to one of the options, this option will have a higher choice probability.

Another theory of preference reversals is provided by Decision Field Theory (Busemeyer & Townsend, 1993), which was developed as a general theory of the process of deliberation and accumulation of preference over time in decision making. This neurocomputational model was then extended (Roe, Busemeyer, & Townsend, 2001) to explain contextual preference reversals. The model is based on a connectionist network that accumulates preferences for each option over time as the decision maker’s attention switches, stochastically, between the different options and their features. Roe et al. (2001) argue that the properties of this accumulation process reveal how the attraction effect arises as a consequence of computing values from differences. Option *B* wins over *A* because it has a bigger relative value to the decoy *D* than does *A* (Busemeyer & Townsend, 1993; Roe et al., 2001).

In Multialternative Decision Field Theory (MDFT), each option inhibits other options (Roe et al., 2001). The strength of this inhibition is inversely proportional to the distance between the options in the feature space. During the deliberation, the decoy option comes to have a negative valence. Because the decoy dominating option is nearer than the other option, it receives a bigger boost from comparison and is more likely to be chosen. Roe et al. (2001, p. 388) state that attraction effect reversals naturally follow from the extended theory.

Another neuro-computational process model, the Leaky Competing Accumulator (LCA; Usher & McClelland, 2001) is a model of perceptual choice and, as with DFT, it has been applied to the problem of explaining contextual preference reversals (Usher et al., 2008). In the model, a deliberation process involves comparing the feature values of each option with each other option. The calculated differences are then transformed into a preference state via a loss averse value function. Because the nondominating option suffers two large disadvantages (one to the decoy and one to the other option, on the *y*-axis in Figure 1), and the dominating option only suffers one large disadvantage (to the other nondecoy option on the *x*-axis) the nondominating option accumulates less preference and is chosen less often. This explanation is similar to that of Tversky and Simonson’s (1993) context dependent preference. In fact LCA can be seen as a neurally plausible implementation of that model (Usher et al., 2008). In LCA the value function is influenced by loss aversion. Usher et al. (2008, p. 297) argue that their account of preference reversals “demonstrates a limitation of rationality in choice preference.” In other words, it is a side effect of a system adapted to other, or more general, purposes.

Bhatia (2013) reports a process model of choice called the *associative accumulation model* in which a number of effects, including contextual preference reversals, are predicted as a consequence of varying levels of association between a feature and an option. Features that are more accessible, for example, because they have stronger associations with options, are assumed to have a bigger impact on the choices made. The level of accessibility can be influenced by changes to the decision task such as adding more options or changing the salience of existing options. The model’s behavior is consistent with a large range of effects that include the attraction, compromise and similarity effects as well as a gain-loss asymmetry relative to a reference point, a range of reference dependent phenomena, the alignability effect and the less-is-more effect. Bhatia’s (2013) associative accumulation model is strongly influenced by MDFT (Roe et al., 2001) and by LCA (Usher & McClelland, 2001) but it extends them by providing a formal account of the whole decision making process (Bhatia, 2013, p. 539).

Trueblood, Brown, and Heathcote (2014) offer a process model of context effects called the multiattribute linear ballistic accumulator (MLBA). Unlike MDFT and LCA this model offers quantitative accounts of the reversal effects. Where evaluations of MDFT and LCA have only been made qualitatively, because of the computational complexity of the simulations, MLBA has an analytic likelihood function that makes it tractable to fit experimental data. MLBA models choice as a race for a threshold between independent accumulators. The speed of each accumulator is determined by a drift rate that is a function of weighted subjective value comparisons, where the weights reflect the amount of attention paid to a particular comparison. It is hypothesized that attention weights should be larger when features are more similar and weights should be smaller when features are easy to differentiate. The model performs impressively when compared against MDFT (see Figure 8 in Trueblood et al., 2014, p. 195).

Wollschläger and Diederich (2012) report a model of attraction, compromise and similarity effects, and the associated decision time. The core assumption of this model is that information is sampled and used to increment counters. Each option has two counters, one for positive information and one for negative information. A random walk of the tree of possible states, where each state is a vector of positive or negative information counters for each option, is used to simulate the decision making process in the standard contextual decision making conditions. The model successfully predicts a range of the known phenomena. Unlike DFT and LCA, inhibition is not required to explain these phenomena. An advantage of the model is that it generates closed form predictions for choice proportions and decision time.

Other models suggest that the attraction effect might be the consequence of low level neural information processing constraints, such as firing rates. It is possible that relative estimates of value may be a consequence of adaptation of neuronal firing to optimize sensitivity across large ranges of value (Seymour & McClure, 2008). In computational neuroscience, Soltani et al. (2012) have proposed a model in which stimuli are normalized so as to be distinguishable by neurons that have a firing rate of between 0 and a few hundred spikes per second. Without normalization, neurons would not be able to represent the range of experienced values. Soltani et al. (2012) show that this neural constraint can lead to preference reversals. An important contribution of this work is that it takes known facts about constraints on the operation of neurons and works through their implications for choice behavior.

In summary, contextual preference reversals have been taken as strong evidence that human decision making processes do not conform to key axioms present in normative theories of rational choice. This observation has led to a prevalent view that preference reversals indicate that value maximization approaches cannot be used to explain behavior (Tversky & Simonson, 1993). Further, influential process explanations of contextual preference reversals suggest the effect is a consequence of cognitive-neural information processing mechanisms (Roe et al., 2001; Usher et al., 2008). Preference reversals, according to these explanations, are an outcome of a bounded system failing to generate the normatively rational solution. In contrast, in what follows, we argue that the decoy provides information that correctly implies that the dominating option has the highest expected value. We demonstrate that a rational analysis can explain *why* people exhibit a range of preference reversal types, including attraction, compromise, similarity, and phantom. Thereby, we provide an analysis of why neural cognitive-neural information processing models *should* exhibit these effects.

## A Model of Computationally Rational Choice

We assume that the decision problem in a preference reversal experiment is an expected value maximizing choice between options, each of which is represented by a set of features, and where the expected value of each option is a function of its features. Here we focus on a task in which a choice must be made from a set of gamble options as in Wedell (1991), each of which is sampled from a distribution of possible options. Each option has a probability feature *p* and a value feature *v*.

The model makes a pair of observations of the options presented in the experiment. One observation is a noisy calculation of the model’s own subjective expected utility. The other observation is a noisy encoding of the partial order of the features of the options, here probabilities and values. (The order of the observations in the model is not important.) We refer to the first observation as the *calculation* observation and the second as the *ordinal* observation. We assume that these two observations are subject to partially uncorrelated noise. This is based on the assumption that the two observation processes are not identical—that they need not operate at the same time or on precisely the same perceptual input. (The theory does not commit to the mechanistic sources of the noise.) Having made the observations, the model chooses the option with the highest expected value given these two noisy observations.

**Figure 2 fig2:**
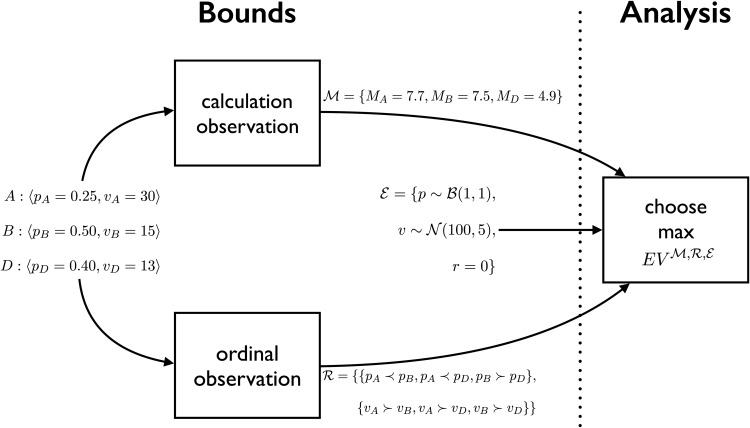
Given a choice task, the theory of bounds (to the left of the dotted line) is that people make two noisy and partially independent observations of the task; one is a noisy, and possibly biased, calculation of subjective expected utility (SEU) and the other a noisy ordinal observation. The order of the observations does not matter. In the example in the figure the observation of *M*_*B*_ is without error but the observation of *M*_*A*_ and *M*_*D*_ have been affected by noise; the observation of R is without error. The analysis (to the right of the dotted line) chooses the option with maximum expected value given the observations M, R and the environment E.

The model is illustrated in Figure 2 and a corresponding formal description is given below. Both are illustrated with a choice among three options, each defined by probability and value features, but the model is easily generalizable to other numbers of options, and other mappings from features to expected value.

1 Each option *i* ∈ {*A, B, D*} is a gamble specified by a 〈probability, value〉 pair.[Fig-anchor fig2]
2 The environment distribution E is the distributions of probability and value. The probabilities *p* are sampled from a distribution with range [0; 1] and the values *v* are sampled from a distribution with a defined central tendency and spread. Here we assume that the probabilities are β distributed (with shape parameters *a* and *b*) and the values are Gaussian distributed (with parameters μ and σ_*val*_) but the model is not committed to these particular distributional assumptions. The model allows for a correlation *r* between *p* and *v*. In general, these distributions allow the modeling of the distributions of probability and value in the adaptation environment.
  p~B(a,b) 1
  $$v\;\sim \;N(\mu ,\sigma_{val}^{2})$$ 2
  r=corr(p,v) 3
3 The calculation observation is $M=\{M_{A},\;M_{B},\;M_{D}\}$ where:
  $$M_{i}=p_{i}^{\alpha }\times U(v_{i})+E,\;E\sim N(0,\sigma_{calc}^{2})$$ 4
  The probability *p* is weighted by an exponential parameter α. The calculation of *M*_*i*_ is assumed to be subject to unbiased Gaussian noise with standard deviation σ_*calc*_. This *calculation noise* represents all of the noise experienced through observing probabilities and values and calculating each *M*_*i*_. The calculation produces a noisy, and possibly biased, estimate of the model’s own subjective expected utility. If α = 1 and *U*(*v*) = *v* then the observation $M_{i}$ is an unbiased but noisy observation of the expected value.
4 The ordinal observation is a set R of partial orderings, one for each feature type. To represent pairwise order relations we define a function *f* which, subject to a small probability of error *error*_*f*_, maps pairs of real numbers to a member of the set O={≺,≡,≻}, where two probabilities are defined as equal if their magnitudes are within tolerance τ_*p*_ and two values are defined as equal if their magnitudes are within tolerance τ_*v*_. The probability of ordinal error is the probability that each order relationship is chosen uniformly randomly from O. For probabilities, the function f is defined as
  $$f(p_{i},p_{j})=\{\begin{array}{l} ≺,\\ \equiv ,\\ ≻, \end{array}\;\begin{array}{l} \text{iff}\;p_{i}<p_{j}-\tau_{p}\\ \text{iff}\;\left|p_{i}-p_{j}\right|\;\leq \;\tau_{p}\\ \text{iff}\;p_{i}>p_{j}+\tau_{p} \end{array}$$ 5
  The set R is then the noisy encoding of the partial orderings of probabilities and values:
  $$\begin{array}{l} R=\{\{f(p_{A},p_{B}),f(p_{A},p_{D}),f(p_{B},p_{D})\},\\ \;\;\;\;\;\{f(v_{A},v_{B}),f(v_{A},v_{D}),f(v_{B},v_{D})\}\} \end{array}$$ 6
5 The expected value of each option *i* given the observations is:
  $$EV^{M,R,E}(i)=E(EV(i)|M,R,E)$$ 7
  $$\;=E(p_{i}\times v_{i}|M,R,E)$$ 8
  where we denote the dependence on the observations and environment with the superscript M, R, E. $EV^{M,R,E}(i)$ is the model’s best estimate of *EV*(*i*) given a particular observation of M, R in environment E. In Bayesian terms, $EV^{M,R,E}(i)$ is the posterior estimate of expected value given the observations.
6 The expected values of all three options are compared and the model chooses the option *i* with the largest expected value:
  $$i^{*}=\underset{\;i\in A}{\text{arg}\;\text{max}}EV^{M,R,E}(i)$$ 9

In summary, the model parameters are:

1 Parameters that define the environment distribution E. These are the shape parameters *a* and *b* of the β distributed probabilities; the mean μ and variance σ_*val*_^2^ of the Gaussian distribution of values; and the correlation *r* between the probability and value. (When we fit the model to data we use the predictive *t* distribution to model the distribution of values in the experiment.)
2 The calculation noise σ_*calc*_^2^ and probability weighting parameter α associated with the calculation observation M.
3 The probability of ordinal error *P*(*error*_*f*_) associated with determining feature ordinals R, and the ordinal tolerances. The tolerances were set to the following values for all analyses presented in the article: τ_*p*_ = 0.011 and τ_*v*_ = 1.1.

In the following sections we explore the consequences of the free parameter settings for the preference reversal rate predicted by the model.

## Results: Deriving Implications of the Theory

All predictions reported in the following sections were generated using a numerical simulation that had the following stages.

• The simulation first constructed, via Monte Carlo sampling, a tabular value function that mapped observations to expected value. It sampled many tasks from the environment distribution E, made M, R observations, and computed conditional expected values. An observation was a pair <M,R> and expected value was a triple $<EV^{M,R,E}\left(A\right),\;EV^{M,R,E}\left(B\right),\;EV^{M,R,E}\left(D\right)>$ (see Definition 7). These conditional expected values were computed by taking the average of the true expected values for the observed sample tasks that met the condition. One to fifty billion samples were used to construct the value function tables in the simulations reported in the article. As there are three levels of feature ordinal (greater, less, and equal) and six relationships between features (Definition 6), there are 3^6^ = 729 possible ordinal observations R. The three calculation observations M were binned into 120 levels to give 120^3^ = 1,728,000 possible observations M. There were, therefore, a total of 1,259,712,000 possible combined observations.
• Once the value function had been constructed it was used to generate choice predictions for tasks (option sets) of interest. These tasks were either tasks sampled from E, or they were the Wedell (1991) tasks, or they were task types, such as compromise and similarity, that were not studied by Wedell (1991) but were generated as variants of the Wedell tasks. Unless otherwise stated, the predictions for each task were generated by sampling 1 million observations. For each observation, the expected values were computed (using the tabular value function), and the option with the highest conditional expected value was selected. (The C^2+^code and executable are available online at http://www-personal.umich.edu/rickl/)

### Demonstration of Expected Value Maximization

**Figure 3 fig3:**
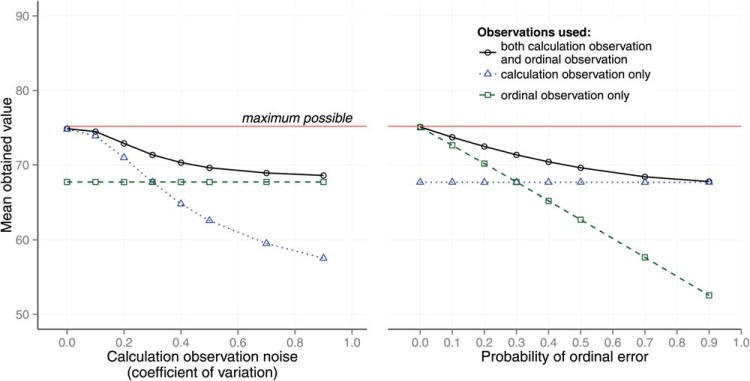
The expected value of choice against coefficient of variation for the calculation error (left panel) and coefficient of variation for the probability of ordinal error (right panel). The different lines are the expected values for the following models: (a) both a calculation observation and an ordinal observation, (b) only a calculation observation, and (c) only an ordinal observation are provided. See the online article for the color version of this figure.

To support the claim that it is rational to make use of ordinal observations, we randomly generated three-option tasks from an environment in which options had a probability feature *p* and a value feature *v* (10 million tasks were sampled for each point in the plot and each task was observed once). The probabilities *p* were β distributed with parameters (*a* = 1, *b* = 1). The values *v* were normally distributed with parameters ($\mu =100,\;\sigma_{val}=5$). The correlation *r* between *p* and *v* was 0 and the probability weighting parameter was α = 1. The maximum value to the model was 75.69. We compared the value of choices made by the simulation described above to the value of choices made by a model that only made use of calculation observations and to the value of choices made by a model that only used ordinal observations. These comparisons are illustrated in Figure 3. [Fig-anchor fig3]

Figure 3 shows 2 panels. In each panel the true expected value of the choice is plotted against noise level. In the left panel the expected value is plotted against calculation noise σ_*calc*_. In this panel, when σ_*calc*_ = 0 the combined observation model observes calculation with perfect acuity and achieves the maximum value from its choices. It does so by virtue of the fact that without noise and with α = 1 the calculation observation observes expected value, which is why the calculation only observation does as well at σ_*calc*_ = 0. As noise in the calculation increases the value received when only using calculation observation diminishes. However, as this happens, the value of choices made by the combined calculation and ordinal observation model, and, therefore, the expected value, diminishes less slowly because noise on ordinal observations is not increasing.

In the right panel, expected value is plotted against the probability of a feature order error *P*(*error*_*f*_). In all three models a constant calculation noise was used (σ_*calc*_ = 30), corresponding to a coefficient of variation of 0.3. All other parameters were unaltered.

### Attraction Effects

In this section we test whether the theory predicts the preference reversals effects observed in Wedell (1991). Wedell’s experimental design has the virtues of systematically varying the decoy position, and using gambles, so that there is an independent principled basis for mapping from the features of each alternative to expected value. We described this study briefly above and expand the description here.

In each of the experimental conditions there were three options {*A*, *B*, *D*}. In one condition the decoy was close to option *A* and in the other condition it was close to *B*. We refer to the option that is proximal to the decoy as the *target* and the other option as the *competitor*. (Terminology varies in the literature.) Option *A* always had a higher probability and option *B* a higher value. Each participant made two choices for each pair of gambles (one choice for each decoy position).

**Figure 4 fig4:**
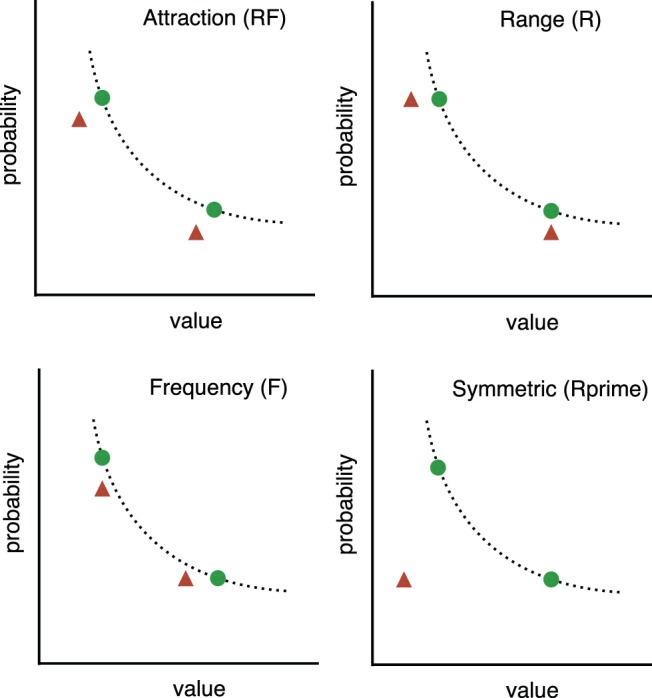
Option positions (R, F, RF, and Rprime) used by Wedell (1991). The dotted line represents the line of equal expected value on which two of the three options sit (green circles). The third option is the decoy (red triangle) and it is in one of two positions in each condition. Its position varies according to condition but it is always dominated by one of the other two options on at least one feature dimension (probability or value). See the online article for the color version of this figure.

In the first two experiments reported by (Wedell, 1991), four positions for the decoy relative to the target were tested. These are shown in Figure 4. The Range decoy (R), Frequency decoy (F), Range-Frequency decoy (RF), and R’ (Rprime decoy) were each set to test a different hypothesis. A preference reversal effect was, as expected, observed in the asymmetric conditions (R, F, and RF) where the decoy is positioned closer to one option than to the other. Furthermore, as expected, the effect was not observed in the symmetric condition (Rprime), where the decoy is dominated by both *A* and *B* on the value dimension. Rprime is an important control and shows that merely introducing any decoy is not sufficient to cause preference reversals (Wedell, 1991). The probabilities and values used to model the R, F, RF and Rprime tasks were those used by (Wedell, 1991). [Fig-anchor fig4]

**Figure 5 fig5:**
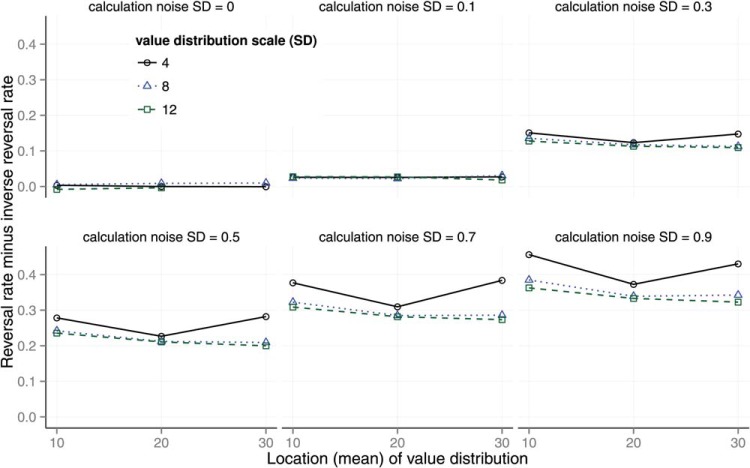
Reversals minus inverse reversals for the RF decoy against the predictive location of the distribution of value *v* in the environment for multiple levels of calculation observation noise (1 level in each panel) and for levels of predictive scale (the lines in each panel). See the online article for the color version of this figure.

The theory was tested by generating predictions using the numerical simulation described above. The effect of calculation noise on preference reversals is shown in Figure 5. The *y*-axis shows the reversal rate minus the inverse reversal rate. The reversal rate is the proportion of trials on which participants selected *A* when the decoy was at *A* and selected *B* when the decoy was at *B* for matched trials that used identical gambles for *A* and *B*. In other words, the reversal rate is the rate at which participants selected the decoy dominating option in both of a pair of matched trials with the same *A*, *B* features but different decoy positions. The inverse reversal rate is the rate at which participants selected *B* when the decoy was at *A* and *A* when the decoy was at *B*. The reversal and inverse reversal rate were reported by Wedell (1991). The inverse rate acts as a control for random variation. The parameters of the model were set as follows. The shape parameters of the β distributed probabilities *p* were set to the maximum likelihood values of (*a* = 1, *b* = 1) given Wedell’s task distributions. The other parameters were set to the following values: $\left\{P(error_{f})=0,\;r=0,\;\alpha =1,\;U(v)=v\right\}$.[Fig-anchor fig5]

There are six panels in Figure 5, each representing the effect of one level of noise on the calculation observation σ ∈ {0, 0.1, 0.3, 0.5, 0.7, 0.9}. Each panel shows the reversal rate for different levels of the location and scale of the environment distribution for feature values *v*. The distribution of *v* used by Wedell was fitted with a scaled, shifted *t*-distribution with *location* = 19.60, *scale* = 8.08, and *df* = 100.

The results in Figure 5 show that preference reversals are predicted as long as calculation noise is nonzero. When calculation noise is zero (top left panel), the model predicts no preference reversals. The panels show how the predicted reversal rate is moderated by calculation noise and by the expected location and scale of the *v* distribution. Most noticeably, increasing calculation noise increases the preference reversal rate.

**Figure 6 fig6:**
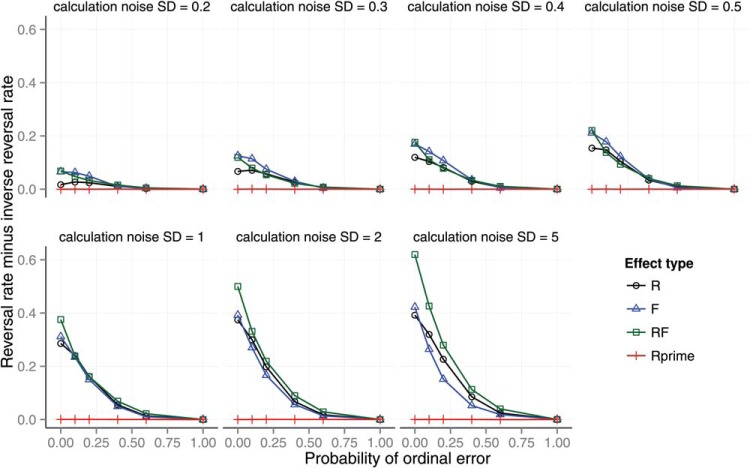
Reversals minus inverse reversals against the probability of an ordinal error for different levels of calculation noise. See the online article for the color version of this figure.

Across seven panels, Figure 6 shows the effect of calculation error and ordinal observation error on preference reversals in each of the four Wedell conditions. The preference reversal rate increases as calculation error increases (across panels) and decreases as ordinal observation error increases (within panels). We also tested the effect of probability weighting on preference reversals (see Appendix A) and the effect of the distance of the RF decoy from the target (see Appendix B).[Fig-anchor fig6]

**Figure 7 fig7:**
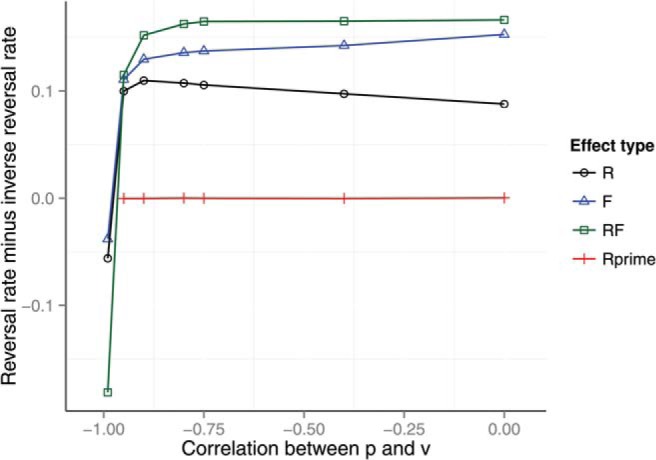
The effect of a negative correlation between *p* and *v* on preference reversals when σ_*calc*_ = 0.4. See the online article for the color version of this figure.

Figure 7 shows the effect of a negative correlation *r* < 0 between *p* and *v* on preference reversals when σ_*calc*_ = 0.1 and α = 1. We investigated the effect of a negative correlation as it is plausible that probability and value are negatively correlated in human experience (Stewart, Chater, & Brown, 2006). As expected, the preference reversal rate is lower with high negative correlation between *p* and *v* than with correlation nearer to zero. In the figure it can be seen that the effect of negative correlation is fairly flat for *r* > −0.9. For *r* < −0.9 the preference reversal rate reduces rapidly until at *r* = −1 the rate become negative. This is because at *r* = −1 all options have the same expected value and decoys are selected at the expense of the target. In summary, the analyses in this section have shown that the model *predicts* preference reversals as long as the calculation observation is noisy and as long as the correlation between *p* and *v* is greater than about −0.9.[Fig-anchor fig7]

#### Model fitting

In this section we report the best fit that we have obtained of the model to the Wedell (1991) effect sizes. First, the scale and location of the value distribution were set to the scale and location of the Wedell task distribution. The probability distribution parameters were held constant at *Beta*(*a* = 1, *b* = 1) and the correlation parameter was set to *r* = 0. No further adjustments to these parameters were made in the following fits to the human data.

Next the values of the calculation noise, ordinal noise and α parameters were adjusted so as to fit the model to the Wedell (1991) data. These parameters were adjusted so as to minimize the Root Mean Squared Error (RMSE) between model and data for the reversal rate, inverse-reversal rate, decoy rate and the difference between the reversal and inverse reversal rate. (An inverse-reversal occurred when participants favored the option that did not dominate the decoy.) The fitted values were: calculation noise σ_*calc*_ = 0.35, ordinal error *P*(*error*_*f*_) = 0.1, and probability weighting parameter α = 1.5. The average RMSE was 1.2 percentage points. The decoy rate of the best fitting model was 2%.

**Figure 8 fig8:**
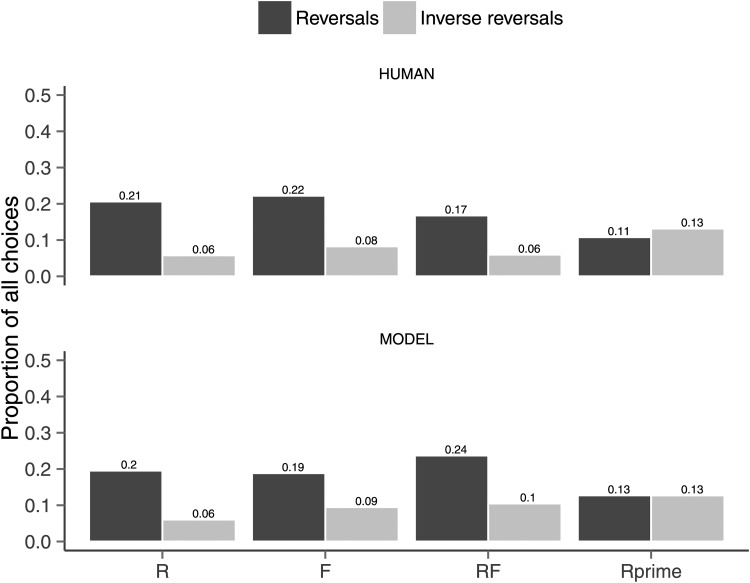
Reversal effects for model fit (bottom) and data from Wedell, 1991 (top). The bar graph shows the reversal and inverse reversal effects for the four major conditions of the Wedell experiment.

Figure 8 contrasts the fitted preference reversals of the model to the human data. The top bar graph of the figure shows the human reversals and inverse reversals for each of the Range (R), Frequency (F), Range-Frequency (RF), and Rprime (R’). It shows that people exhibited more reversals than inverse reversals in all conditions except Rprime. Wedell (1991) report that these results were significant. The model effects are shown in the bottom bar graph of Figure 8. More important, the model fit captures the absence of an effect of Rprime (Wedell, 1991; see also Figure 6).[Fig-anchor fig8]

In addition, we fitted the model to the inverse reversal rate only. With this model the calculation observation noise, the ordinal observation error and α were adjusted to fit the model’s inverse reversal rates to the human data. The reversal rates were not fitted for this analysis and are, therefore, predictions. The model predicted preference reversals in R, F, and RF conditions and it predicted the absence of an effect in Rprime. However, effect sizes were larger than those observed in humans.

#### Discussion

The model described above predicts the qualitative preference reversal effects observed by Wedell (1991). It does so as long as there is some uncertainty in the observation of *p*_*i*_ × *v*_*i*_ and as long as there is some partially independent observation of feature ordinals. Further, it does so in Wedell’s R, F, and RF conditions and it predicts the absence of an effect in the symmetric (Rprime) condition, which provides a control.

The reason that the model predicts these contextual preference reversal effects is that making preference reversals, when observations are noisy, is an implication of expected value maximizing choice. The model shows that there is nothing irrational about preference reversals given these assumptions. In fact, there is no sense in which making preference reversals reveals a change in preferences, once it is understood that the choice tasks in these experiments are choices with uncertainty about expected value. Given this uncertainty and the availability of partially independent ordinal observation, the expected value of options is defined by the analysis provided above. We offer further explanation on the role of ordinal observation in contextual choices below.

### Compromise, Similarity, and Phantom Effects

**Figure 9 fig9:**
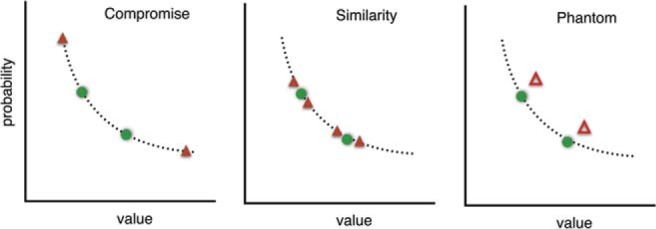
Option positions for compromise, similarity and phantom choice conditions. In the compromise and similarity tasks only the two green-circle options and one of the red-triangle options are available. In the similarity task there are four possible decoy positions all of which have the same expected value as the other two options. In the phantom case the decoy option is not available for choice and the phantom option is positioned so as to dominate one of the other options. See the online article for the color version of this figure.

In this section we use the same theoretical assumptions to predict the effects of the compromise, similarity, phantom and inferior but nondominated decoy positions. All but the last of these decoy positions are illustrated in Figure 9. The last, the inferior but nondominated decoy position, was set between the compromise and F position (see Figure 4) so that it was below the line of equal expected value and not dominated by either option. To model this scenario, the ordinal observation was made with the decoy present (as above) but the model was not permitted to select the decoy (unlike above). The change is only required to model the task and involves no changes to the theoretical assumptions.[Fig-anchor fig9]

In the left panel of Figure 9, the two possible positions of a compromise decoy (red triangle) are shown. All three options in a task have the same expected value and the decoy is known to increase human preference for the option that lies between the other two on the line of equal expected value. In the middle panel, the four possible positions of the similarity decoy position are shown. Again, only one of these positions is used in any one task. Two of the positions are outside of the other two options and two are in between. In all cases, the similarity decoy is very close to the target option and has the same expected value. The phantom positions are shown in the right most panel. These are illustrated with red triangles that are open to indicate that they cannot be chosen.

**Figure 10 fig10:**
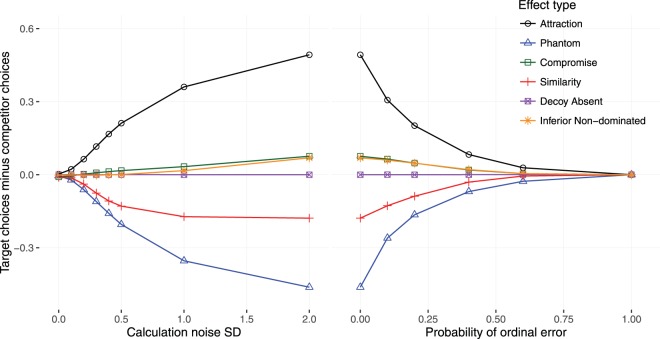
Predicted proportion of target minus competitor selections for five preference reversal conditions against calculation noise (left panel) and ordinal noise (right panel). The Decoy Absent condition is the control and represents the two choice task. All parameters, other than those manipulated, were held constant (see text for details). See the online article for the color version of this figure.

The predictions of the model in the similarity, compromise, phantom, and inferior nondominated conditions are shown in Figure 10. Each panel shows the consequences of different levels of one of the noise parameters when the other parameters were set to: $\left\{location=19.60,\;scale=8.08,\;Beta(a=1,\;b=1),P(error_{f})=0,\;\sigma_{calc}^{2}=2.0,\;r=0,\;\alpha =1\right\}$. (The *location* and *scale* of the t-distribution of value are the maximum likelihood values given Wedell’s materials.) The left panel represents the effect of calculation noise and the right panel represents the effect of ordinal error. The panels show the predicted number of target selections minus the number of competitor selections, where the target is the option that is proximal to the decoy. This measure is a measure of the preference for the target over the competitor. It is used instead of the reversal minus inverse reversal measure used in the previous section because it is the measure used in the studies reported by Trueblood et al. (2013) and others. For comparison purposes the model also shows the number of selections of target minus competitor for the attraction decoy and for when the decoy is absent. Each line in Figure 10 is for one of the decoy positions. This is an average of two positions for compromise and phantom decoys and the average of four positions for the similarity decoy.[Fig-anchor fig10]

The left panel of the figure shows that irrespective of the level of calculation noise, if the decoy is absent, then each of the two options are predicted to be selected about half of the time. In contrast, as calculation noise increases then there is a predicted positive effect of the attraction and the compromise decoy on target selections. The compromise decoy prediction is in the same direction as that observed by Trueblood et al. (2013). The formal explanation for the compromise effect is in the theoretical assumptions described above. Informally, the compromise effect is a consequence of the expected values of the three options given the ordinal observation and utility observations. Imagine a set of randomly sampled option triples given the distributional and correlation properties of the environment. The compromise condition is a particular subset of this distribution in which *p*_*A*_ > *p*_*B*_ > *p*_*D*_ and *v*_*A*_ < *v*_*B*_ < *v*_*D*_. Given the standard definition of the expected value, then the calculations reported in Figure 10 show that the expected value of option B is greater than the expected value of the other two options more frequently than vice versa. However, the extent to which this outcome is true is dependent on the environmental parameters; a further analysis of scale and location of value is provided in Appendix C. In addition, the size of the compromise effect is moderated by the distance of the compromise option from the target option; see Appendix D.

Figure 10 also shows the predicted effect of the similarity decoy and the phantom decoy. The effect of the similarity decoy was found to be the same for all four pairs of the four similarity decoy positions and the figure shows the average. When ordinal observation noise is low, these decoy positions cause the model to predict a reduced proportion of selections of the option that is close to the decoy, which is consistent with the observed human behavior (Maylor & Roberts, 2007; Trueblood et al., 2013; Tversky, 1972).

Further investigation of the results revealed that the similarity prediction is because of *substitution*; when the similarity decoy is present then choices that would otherwise have gone to the target are shared between the target and the decoy, thereby reducing the number of target selections relative to the situation where the decoy is absent. Substitution effects have been discussed in consumer choice, and Evers and Lakens (2014) provide evidence that they explain apparent similarity effects in simple categorization tasks. As a consequence of the substitution of decoy for target, the model predicts high rates of decoy selection in the similarity condition. This prediction is supported by Trueblood (2012) where the similarity decoy selection rate was about 20% (Trueblood (2012), p. 966; Figure 3a).

The predicted phantom effect is only present when there is calculation noise (left panel of Figure 10). As with the similarity effect, the predicted phantom effect *reduces* the number of target selections. This prediction is consistent with some other models, but not all. The fact that existing models offer inconsistent phantom predictions has been noted before (Scarpi & Pizzi, 2013). Our model’s phantom prediction is consistent with the results of a study of lottery choice reported by Soltani et al. (2012). In the Soltani study, participants were presented with three monetary gambles on a screen for 8 s and at the end of that period one of the gambles—the phantom decoy—was removed and the participant selected one of the remaining gambles within a 2 s period. The phantom decoy dominated one of the other options. Soltani et al. (2012, p. 4) found that the phantom decoys decreased the selection of the target gamble.

While the prediction of our model is consistent with Soltani et al. (2012), both prediction and data are inconsistent with the phantom effects observed by (Pettibone & Wedell, 2000, 2007). Pettibone and Wedell (2007) studied the effect of five phantom positions in two experiments. The tasks involved consumer choices such as a choice between computers on the basis of two dimensions, say memory and speed. The five positions dominated one of the other options on either one dimension or both and were either closer or further from the target option. The Pettibone and Wedell (2007) tasks did not involve choices between gambles, unlike Soltani et al. (2012). In 4 out of 5 of the decoy conditions in Experiment 1 of Pettibone and Wedell (2007) a significant positive effect of the phantom decoy was observed; the phantom decoy *increased* the proportion of target selections. The same was true in 2 out of the 5 decoy conditions in Experiment 2 of Pettibone and Wedell (2007); a significant effect was absent in 3 of the 5 conditions and, on average, it was diminished in magnitude. Experiment 2 used a within participant design and further analysis was conducted to understand the individual differences. Pettibone and Wedell (2007) split the participants into three groups, a positive group (*N* = 79), a low group (*N* = 156), and a negative group (*N* = 27) on the basis of arbitrary cutoffs in the individual phantom effect size. What was interesting was the extent to which there was individual variation in the effect of the phantom decoy. Of the three groups, only the group of 79 of the 262 participants generated a reliable positive phantom decoy effect in all five of the decoy conditions. The result for the largest group of participants (*N* = 156) were either mixed or absent (there was little compelling evidence for a positive or negative phantom effect). The result for the relatively small group of participants (*N* = 27) was a negative phantom effect (as predicted by our analysis). Although this was the smallest group, the negative effect was present in all five of the phantom conditions and the magnitude of the negative effect for this group was about the same size as the magnitude of the positive effect exhibited by the positive group.

In summary, our analysis of the effect of the phantom decoy suggests that the average behavior of the participants studied by Soltani et al. (2012) was rational; by making ordinal observations relative to an unavailable option suppresses the selection of options dominated by a phantom, these participants were behaving in a way that is consistent with an observer that seeks to maximize the expected value of selected gambles given noise. Similarly, the individual differences analysis provided by Pettibone and Wedell (2007) suggests that at least a small group of their participants made rational, expected value maximizing decisions, when presented with consumer choice triads that included a phantom decoy.

### Time Pressure Effects

**Figure 11 fig11:**
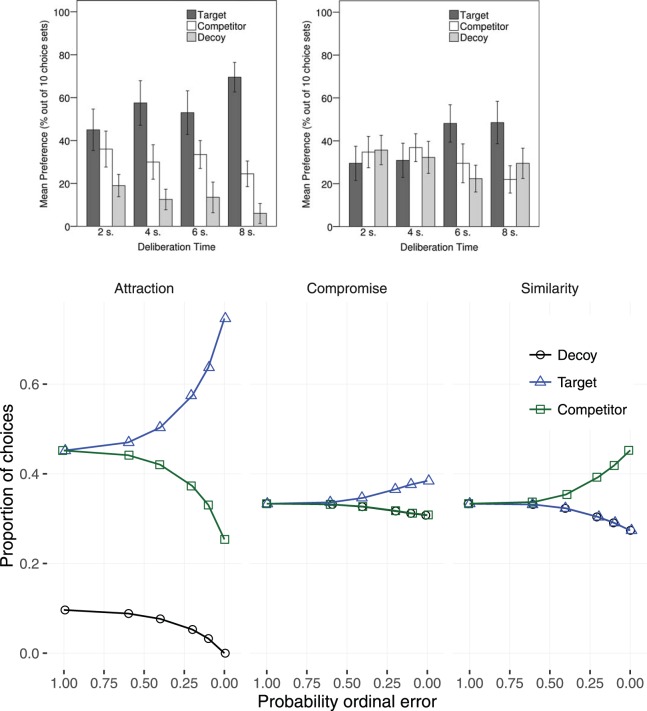
Top panels: Observed effects of time pressure on attraction and compromise effects (similarity not tested) for human target selections, from “Testing the Effect of Time Pressure on Asymmetric Dominance and Compromise Decoys in Choice,” by J. C. Pettibone, 2012, *Judgment and Decision Making, 7*, pp. 516–517. Copyright 2012 by the Society for Judgment and Decision Making. Bottom panels: Predicted effect of ordinal observation error on preference reversals (*x*-axis reversed). From left to right: attraction, compromise and similarity decoys. See the online article for the color version of this figure.

A number of studies suggest that the rate of contextual preference reversals diminishes as time pressure increases (Dhar, Nowlis, & Sherman, 2000; Pettibone, 2012; Simonson, 1989; Trueblood et al., 2014). In particular, Pettibone (2012) has shown an effect of manipulating the time available to view options on the compromise and attraction effect. In a between-participants design participants were given 2, 4, 6, or 8 s to view a three alternative, two attribute, choice set and then asked to choose. The results of Pettibone’s study are presented in the top panels of Figure 11. The top left panel is for the attraction decoy positions and the top middle panel for the compromise positions. In both figures lower time pressure is to the right on the *x*-axis and higher time pressure to the left. As time pressure increases—right-to-left—it can be seen that the differences between the target, competitor, and decoy selection proportions diminishes. It can also be seen that the decoy option is selected more frequently in the compromise condition than in the attraction condition. The effect of time pressure on the human similarity effect was not investigated by Pettibone (2012) and is not illustrated, but has been demonstrated empirically by Trueblood et al. (2014) who found that time pressure diminished the magnitude of the effect.[Fig-anchor fig11]

The model’s predictions, bottom panels of Figure 11, are based on the assumptions that (a) even when there is no time pressure there is some noise in the calculation observation and, therefore, uncertainty about the expected value of the option (this is the same assumption as has been made in all previous analyses in the current article), and (b) the ability of participants to accurately perceive ordinal feature relationships diminishes with time pressure and, therefore, the model parameter *P*(*error*_*f*_) increases with time pressure. Calculation observation error was fitted to Pettibone’s data σ_*calc*_ = .5. This value is substantially higher than the fitted value for the model of Wedell (1991), perhaps reflecting higher uncertainty in Pettibone (2012)’s participant’s estimates of subjective expected value. In the Figure, effect on target, competitor and decoy selections is shown against increasing—right-to-left—ordinal observation error. The attraction and compromise effects are larger at lower ordinal observation error than at higher ordinal observation error. In both model and humans, increased time pressure is accompanied by a decrease in the target selection rate and an increase in the competitor and decoy selection rates.

While Figure 11 shows that the quantitative fit of the time pressure effect is excellent for the attraction and compromise data reported by Pettibone (2012), the results are a less good prediction of the effect of time pressure on the similarity effect reported by Trueblood et al. (2014). However, the time pressure effects reported by Trueblood et al. (2014) were for tasks with very different time profiles (a legal inference task and a perceptual size judgment task) to that studied by Pettibone (2012). Given the differences in the tasks we have not conducted a cross-experiment comparison of the effect sizes. The important point is that the similarity predictions are in the same direction as the human data; time pressure diminishes the effect size.

## The Consequences of Ordinal Observation for Preference Reversals

The analysis above demonstrates that the choice model presented previously in the article can predict human performance on a range of contextual choice tasks. Here, we offer two further analyses so as to help explain why the model predicts preference reversals.

### The Implications of Ordinal Observation for Expected Value

**Figure 12 fig12:**
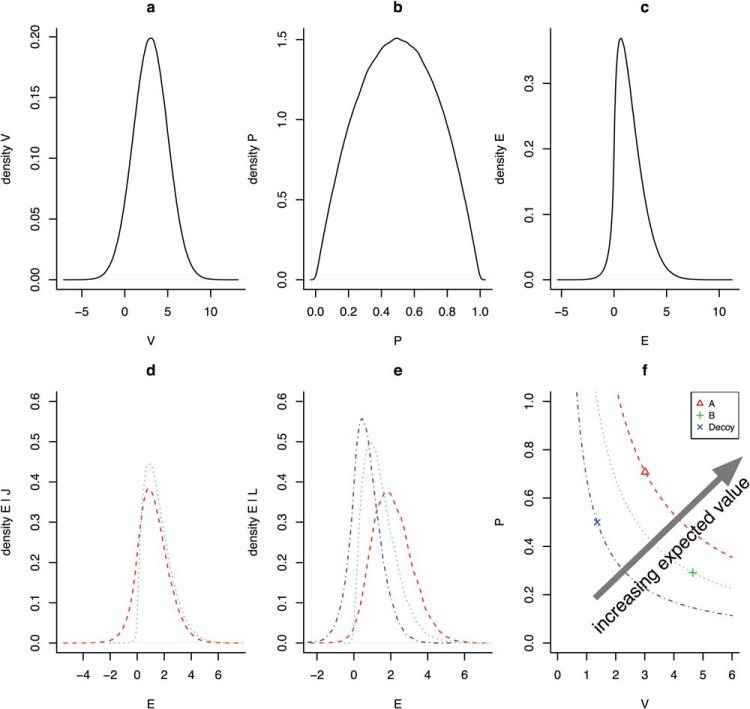
(a and b) Values of *p* for each option were sampled from a β distribution with shape parameters (2, 2) and values of *v* were sampled, independently, from a Gaussian distribution with *mean* = 3, *SD* = 2. (c) The density of expected values, *E* = p × *v*, of options given no constraints. (d) Densities of *E* | *J* for each of two options. (e) Densities of *E* | *L* for the three options that include the decoy. (f) *E* of each of the three options and their equal expected value curves given *L*. *J* and *L* are ordinal constraints specified in the text. See the online article for the color version of this figure.

We have shown that an expected value maximizing model that makes noisy calculation observations and noisy ordinal observations makes choices between options that *is* influenced by the addition of new options, and gives rise to a pattern of preference reversals that is in many cases strikingly similar to the human data. The key to understanding these findings is to understand the implications of ordinal observation for the expected value of choice. An analysis of attraction decoy choice tasks given perfect ordinal observation is provided in Figure 12 where a problem consists of three options *A*, *B*, and *D* and each option is described in terms of a pair of random variables; a random probability *p* and a random value *v*. Figure 12a and 12b represent the densities of these random variables for problems with the reported parametric values and Figure 12c represents the density of the expected values of these options assuming that there are no constraints on their relative value. [Fig-anchor fig12]

Decoy absent choice problems (Figure 12d) are constructed by sampling *p* and *v* values for only two options *A* and *B* and accepting samples for which constraint *J* holds.

$$J=p_{A}\;>\;p_{B},v_{B}\;>\;v_{A}$$ 10

The densities of the expected values of these options are represented in Figure 12d. Notice that there is a small difference in the expected values of *A* and *B*, which derives from the asymmetric contribution of probability and value to expected value. Notice too that the advantage in this case is in favor of *B*; on average *B* is a better choice than *A* in the two choice scenario.

Typically, a constraint is imposed in the decoy present condition of a preference reversal experiment. This constraint can be represented with the following inequalities.

$$L=p_{A}>p_{D}>p_{B},v_{B}>v_{A}>v_{D}$$ 11

*L* captures the fact that *A* must strictly dominate *D* but also that *B* does not dominate, and is not dominated by, *D* or *A* on either dimension. Decoy present problems are constructed by sampling *p* and *v* pairs for each of the three options from the distributions illustrated in Figure 12a and 12b and only accepting problems in which *L* holds. For these cases Figure 12e represents the densities of *E*. It is clear in Figure 12e that, on average, option *A* has a higher expected value than the other options. The preference for *B* in the two choice task has been altered to a preference for *A* in the attraction decoy situation.

In Figure 12f the mean values of *p* and *v* for each of *A*, *B*, and *D* are represented along with 95% confidence intervals (Cis), which are smaller than the plotted points, and lines representing equal expected value.^2^ These values are the average values of *p* and *v* given the constraint *L*. In Figure 12f, it is clear that, given the prior distributions of *p* and *v* when the dominance relationship *L* holds, selecting *A* is expected value maximizing.

The above analysis shows that it can be entirely rational for a person to select a dominating option *A* rather than a nondominating option *B* when a decoy is present, even when there should be an advantage for *B* over *A* when the decoy is absent. The analysis thereby explains the empirical observations of Huber et al. (1982) and also Wedell (1991) as rational choice given uncertainty about the utility of options.

**Figure 13 fig13:**
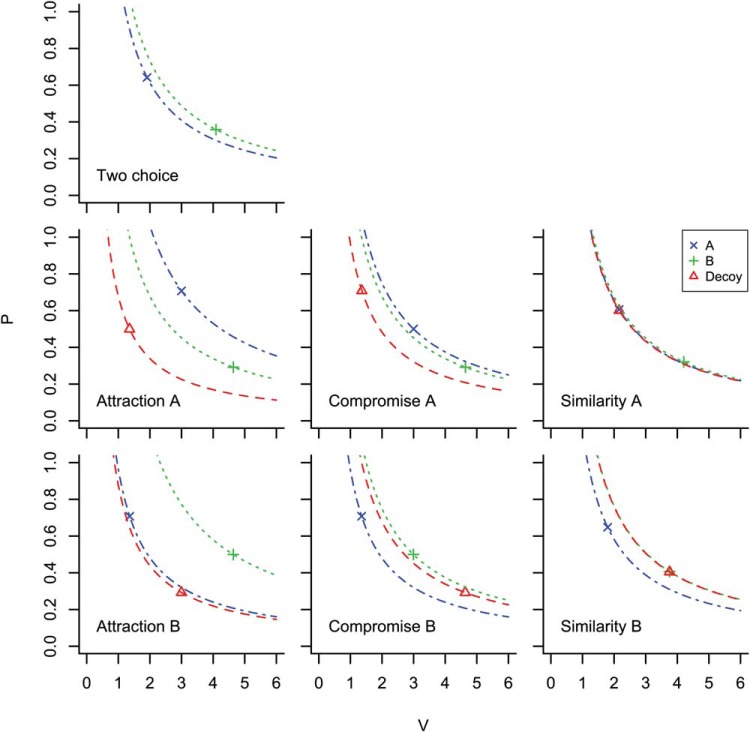
The expected value of each of three options given ordinal constraints on their probabilities and values. The left top panel is for two options, one of which has a higher probability and a lower value. The left middle and bottom panels are for the attraction constraint, the middle column for the compromise constraint and the right column for similarity. See the online article for the color version of this figure.

Figure 13 extends the previous analysis to the compromise and similarity effects. Probabilities and values were sampled from distributions with the same parameters as in Figure 12 and then subsets of options were sampled according to the constraints of the condition (two-options, attraction, compromise, or similarity). The top left panel of the figure shows the expected values of two options that correspond to constraint *J* (see above; one option has higher probability and the other higher value). In the middle panel of the left column, the expected values of the three attraction options, when the decoy is dominated by the high probability option, is shown. This panel shows the same result as that shown in Figure 12. The bottom left panel is also for the attraction condition but here the decoy is dominated by the low probability option. The figure shows the reversal in the order of the expected values of the two superior options when the decoy position changes. The middle panel extends the analysis to the compromise condition. Here the constraint used for the middle column, middle row compromise plot (Compromise A) was:

$$L_{comp}=v_{B}>v_{A}>v_{D},p_{D}>p_{A}>p_{B},|E_{D}-E_{T}|<\delta ,|E_{D}-E_{C}|<\delta$$ 12

where δ represents some small difference to ensure that the decoy has an expected value *E* close to that of both *A* and *B*. Lastly, for one of the similarity conditions, the constraint was,

$$L_{sim}=p_{A}>p_{B},v_{B}>p_{A},|v_{B}-v_{D}|<\delta ,|p_{B}-p_{D}|<\delta$$ 13

[Fig-anchor fig13]

The figure shows which option has the highest expected value changes with decoy position in the attraction (left column) and compromise condition (middle column). In the similarity condition, the decoy is substituted for the target, splitting the target’s share of the choices.

### A Formal Model for Decision Problems With Ordinal Observation

In this section, we demonstrate that preference reversals are also a consequence of expected value maximization given *utility* ordinals. We assume a decision maker that has no a priori knowledge about the utility of presented options. We model this situation with two options, *A* and *B*, with utilities that are random variables, *U*_*A*_ and *U*_*B*_, sampled from the same distribution. Assuming $U_{A}\neq U_{B}$ and in the absence of any other information $p(U_{A}>U_{B})=\frac{1}{2}$.

Given the addition of a third random variable *U*_*D*_, also sampled from the same distribution, where $U_{D}\neq U_{A}$ and a dominance constraint *K* = *U*_*B*_ > *U*_*D*_, then by a simple application of Bayes’ rule we see that $p(U_{B}>U_{A}|K)=p(U_{B}>U_{A}\;\text{and}\;K)/p(K)$. The numerator and denominator can be calculated simply by listing all six possible and equally likely dominance relationships between the three values. In three of these scenarios, namely $\left(U_{B}>U_{A}>U_{D}),\;(U_{B}>U_{D}>U_{A}\right)$ and (*U*_*A*_ > *U*_*B*_ > *U*_*D*_) the constraint *K* holds; consequently, *p*(*K*) = $\frac{3}{6}$. However, in only two dominance relationships, namely (*U*_*E*_ > *U*_*A*_ > *U*_*D*_) and (*U*_*E*_ > *U*_*D*_ > *U*_*A*_), do both *K* and *U*_*B*_ > *U*_*A*_ hold. It follows that the numerator $p(U_{B}>U_{A}\;\text{and}\;K)=\frac{2}{6}$ and that $p(U_{B}>U_{A}|K)=\frac{2}{3}$.

As a result, given a choice between random variables *U*_*A*_, *U*_*B*_, and *U*_*D*_, it will be optimal to prefer *U*_*B*_ over *U*_*A*_ given only the information that *U*_*B*_ > *U*_*D*_. In other words, if *U*_*B*_ *dominates U*_*D*_ then *U*_*B*_ should be preferred over *U*_*A*_. This analysis shows that it is rational for a preference ordering between two options to be influenced by information about the relative value between one of these options and a third option. This analysis holds as long as there is some uncertainty about the value of the options and the distribution of utilities is bounded.

## General Discussion

We have presented an analysis of choice that demonstrates that preference reversals are a consequence of expected value maximization in the face of noisy observations of options. The analysis assumes two observations: one observation is a noisy, and possibly biased, calculation of subjective expected utility for each option and the other is a noisy observation of the partial orderings of features across options. Contextual preference reversals are predicted when the calculation observation is uncertain and when the ordinal and calculation observations are partially independent. Under these conditions an expected value maximizing model will exhibit preference reversals in the attraction, compromise, and similarity conditions. In addition, the analysis showed that rational choice predicts a number of other preference reversal effects, including the (target suppressing) phantom effect, the inferior nondominated effect, the effect of time pressure and the difference in magnitude of the time pressure effect between compromise and attraction conditions.

An implication of the analyses is that people who observe ordinal relationships, subject to noise in their calculation of subjective expected utility, will make better decisions (gain higher value) than people who do not. If a person who reverses preferences in the presence of a dominated option does so in environments where the choice matters, then the extent to which they maximize expected value will be greater than if they do not. It is for this reason that preference reversals are not irrational or illogical; nor are they necessarily a departure from the axioms of rationality as they should be applied to understanding the behavior of computationally bounded minds. On the contrary, under the assumptions of the model presented here, a person who fails to reverse preferences will fail to gain the maximum expected value available to a model that can only make noisy observations of options.

One potential limitation of the analysis is that it says only a little about the underlying information processing mechanisms. The extent to which rational analysis can inform theories of mechanism has been the subject of a recent debate (Bowers & Davis, 2012; Griffiths, Chater, Norris, & Pouget, 2012; Hahn, 2014; McClelland et al., 2010; Norris & Kinoshita, 2010). From one point of view our analysis says only that a rational information processing mechanism should make preference reversals when new options are added to the context. This is a contribution at the level of computational theory (Marr, 1982; Oaksford & Chater, 2007). It is made by studying the environment of cognition, as recommended by Anderson (1990). However, our analysis says more. By virtue of the fact that decisions are bounded by limitations on a person’s ability to calculate the expected value of options, the analysis reveals the rational choice given the hypothesized bounds. In this sense, the analysis shows that preference reversals are *computationally rational* (Howes et al., 2009; Lewis et al., 2014). Specifically, the analysis suggests that under uncertainty induced by the inevitable limitations of biological information processing systems, a mechanism can improve the expected value of choice if it makes and uses ordinal observations.

The analysis of rationality that we have used adopts a methodological optimality approach, as advocated by Oaksford and Chater (1994). While the models make use of optimization to gain their explanatory force, they do not demand that people perform optimizations, nor that they maximize expected value to the extent shown to be possible by the particular account above.

Our analysis shows that preference reversals are a consequence of expected value maximization but not that expected value maximization is required for preference reversals. There are many algorithms for integrating information from multiple sources that would not be optimal but that would generate higher expected value than either the calculation or ordinal observations alone. A bounded information processing system that could only compute approximate estimates of expected value, for example, should still exhibit preference reversals so long as it has some capacity to make ordinal observations. In these circumstances, the use of ordinal observations leads to preference reversals and to better decisions as a consequence.

An implication of this observation is that a wide range of theories of the cognitive mechanisms that predict preference reversals may be rationally adapted to the choice task. DFT, LCA, and Range-Normalization, which offer process explanations of *how* contextual reversals might arise as a consequence of interactions between units in a parallel distributed network (Bhatia, 2013; Roe et al., 2001; Usher et al., 2008) or interactions between neurons (Soltani et al., 2012), may be mechanism theories of rational choice given processing limitations. Further, it is conceivable that the rank dependent mechanisms proposed and reviewed in Roe et al. (2001); Tsetsos, Chater, and Usher (2012); Tversky and Simonson (1993); Usher et al. (2008); or the comparison only models (Stewart et al., 2006; Vlaev, Chater, Stewart, & Brown, 2011), are rational. In other words, making comparisons between features of options, by whatever means, may just be an efficient way for a bounded information processing system to deal with the inevitable uncertainty that attends the noisy integration of features. What the analysis in the current article shows is that, contrary to some views, these mechanisms and strategies may generate rational rather than distorted choices under uncertainty.

Our approach has common purpose with a number of recent contributions that demonstrate that preference reversals can be rational under a range of assumptions (Bordley, 1992; McNamara et al., 2014; Shenoy & Yu, 2013; Trimmer, 2013). As we said, Bordley (1992) argued that individuals have prior expectations about the value of lotteries and if these are taken into account according to Bayesian principles, then preference reversals will follow. He points out that a correct application of expected utility theory—that takes into account priors—can lead to very different decision outcomes than those advertised in much of the decision making literature. Trimmer (2013) shows that violations of regularity and independence can be optimal in an evolutionary context. In two models he considers an animal choosing which herd of prey to attack. The models demonstrate that even options that are never chosen by an animal can be relevant to a decision and it is, therefore, not the case that violations of regularity indicate suboptimal behavior. McNamara et al. (2014) uses a foraging model to show that an animal that is maximizing its rate of food gain can violate transitivity and IIA (Independence from Irrelevant Alternatives). However, unlike in our model, McNamara et al.’s model only demonstrates violations of independence when choices may not be available in the future (as happens in real-world foraging environments).

Shenoy and Yu (2013) also describe a Bayesian model of contextual choice. It is a normative account, following Marr (1982)’s framework, and informed by economic theory, in which the assumptions concern what people believe is fair in the marketplace. In contrast, our model is focused on what is rational given the constraints imposed by cognitive mechanisms. Further, where we focus on the combination of multiple uncertain observations of the task, Shenoy and Yu (2013) assume that uncertainty in posterior beliefs about market conditions contributes to randomness in choice on repeated presentations of the same options (see also Regenwetter and Davis-Stober (2012) for an analysis of choice variability and preference consistency). The essence of Shenoy and Yu (2013)’s explanation is that the introduction of a decoy moves “the indifference line,” which is an estimate of fair value in a perceived market. The essence of our explanation is that ordinal observations allow a decision maker to make improved estimates of expected values. Furthermore, an important difference is our derivation that $p(U_{B}>U_{A}|U_{B}>U_{D})=\frac{2}{3}$ that links the contextual preference reversal phenomena to other decision making behaviors that can be explained with an analysis of the Monty Hall problem (for example, see Chen, 2008; Krauss & Wang, 2003). This analysis (explained above) provides a general demonstration of the rationality of preference reversals.

Our analysis (and the other rational analyses) still faces substantial challenges in showing that all of the empirical phenomena—including some that are explained by existing process accounts—are a consequence of expected value maximization. For example, Bhatia (2013) not only explains contextual preference reversals but also decision making effects such as the less-is-more effect, the alignability effect, and gain-loss asymmetry. In addition, Scarpi and Pizzi (2013) demonstrate empirically that the phantom decoy can either suppress or enhance the proportion of target selections. In their study, they manipulated whether or not a decoy was “known.” In the “known” condition, the participants were told, before selecting an option, that the decoy (the best option) would not be available. In the “unknown” condition, the participants were allowed to choose before the decoy was subsequently withdrawn, resulting in most participants choosing the decoy only to then be told that it is not available. The effect of the phantom was to enhance target selection in the known condition and to suppress target selection in the unknown condition. Our model is not a sequential process model and there is no straightforward means of generating different predictions for known and unknown scenarios. However, these data will have to be addressed in the future.

An additional challenge concerns the computational complexity of the analysis. Our current computational simulation requires billions of simulation trials (one billion take about 20 min on a modern desktop computer) to calculate the expected values of three options and the number of trials required would grow with more options. However, there is no *theoretical* problem posed here. The theory does not need to be generalized to the case of larger option set size; it is well-defined for any set size. However, there are nevertheless two important issues that arise in considering large set sizes. The first concerns the tractability of generating the predictions of the model. For larger set sizes, there is no doubt that more efficient algorithms for approximating optimal solutions will be required. It is beyond the scope of our current work to pursue that now. The second issue concerns whether future empirical shortcomings of the model might be identified and understood to be related to human bounds on integrating observations and managing a large decision space. We think this is also an interesting area to pursue for future work.

What can we now say about Tversky and Simonson’s (1993), and others, rejection of people as value maximizers—a rejection that has in part led to the popular belief that people are irrational (Simonson, 2014)? We have shown that preference reversals are a consequence of value maximization and noisy observations. Our analysis makes the simple assumption, uncontroversial in statistical decision theory, that value maximization given uncertainty should make use of available noisy information to calculate the expected value of options. Value maximization requires making best use of all of the information available according to its precision, and doing so leads to preference reversals. Our analysis not only shows that contextual preference reversals should not be taken as evidence against value maximizing in people. It also shows that preference reversals should be read as evidence that *people are value maximizing* given the limitations of their neural mechanisms—that is, they are computationally rational.

## Figures and Tables

**Figure A1 fig14:**
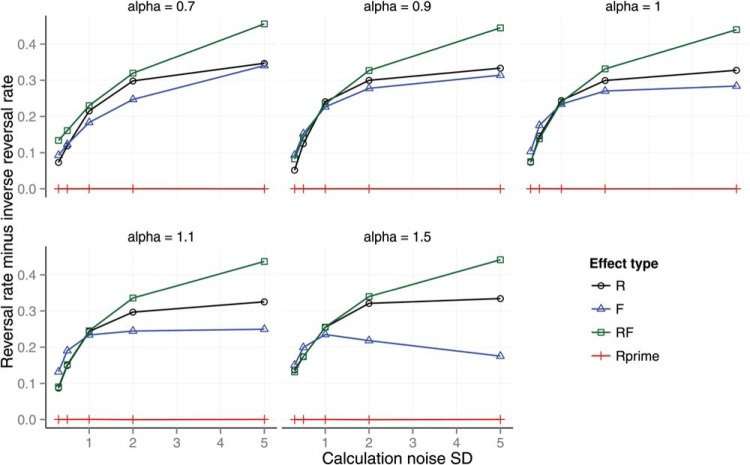
The effect of α and calculation noise on preference reversal rate for the four conditions in the Wedell (1991) studies. See the online article for the color version of this figure.

**Figure B1 fig15:**
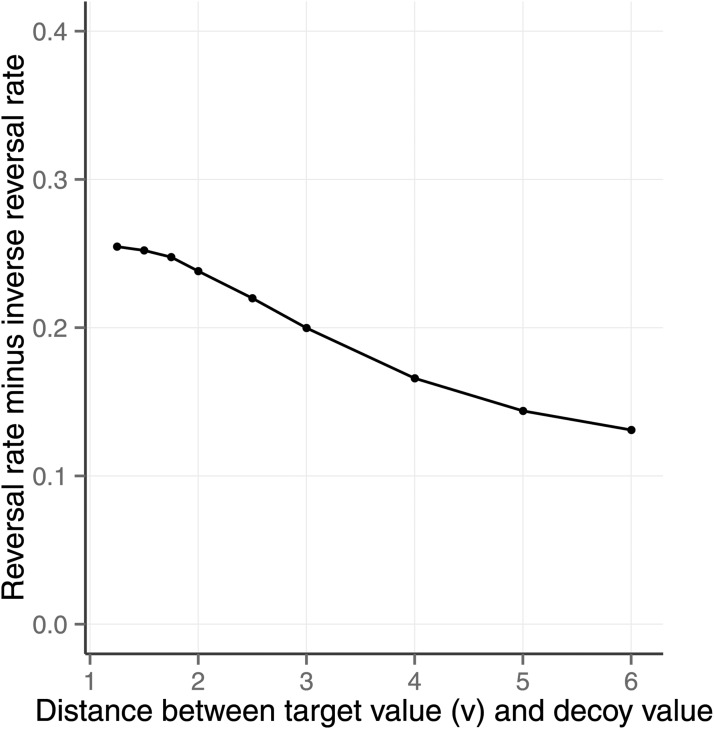
The magnitude of the RF preference reversal effect against the distance of the decoy from the target. The *x*-axis shows the difference between the target value (v) and the decoy value. The probability of the decoy was adjusted with v to maintain the RF decoy position.

**Figure C1 fig16:**
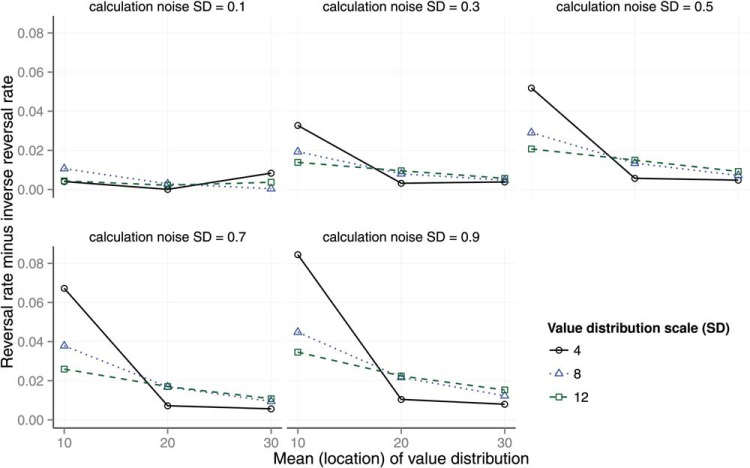
The magnitude of the compromise effect for three levels of the scale of the value distribution against the location of the value distribution. See the online article for the color version of this figure.

**Figure D1 fig17:**
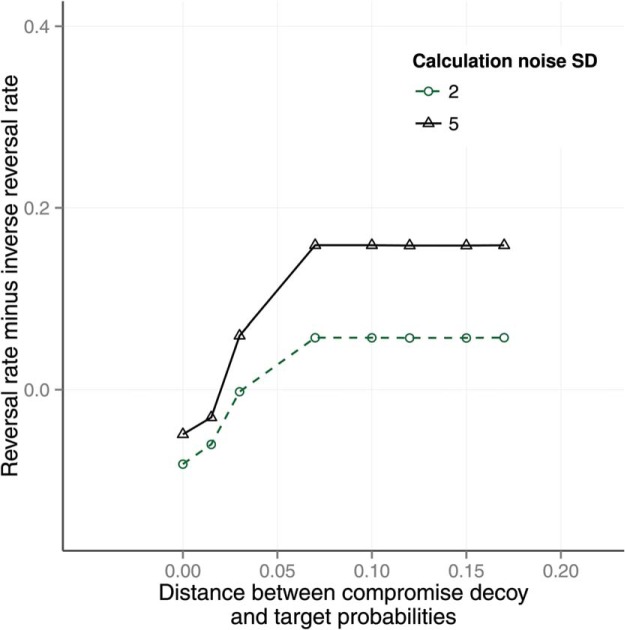
The magnitude of the compromise effect against decoy distance for two levels of calculation noise. See the online article for the color version of this figure.

## Footnotes

1 Shenoy and Yu (2013) was brought to our attention by a reviewer of a previous version of the current article that had been submitted before publication of Shenoy and Yu (2013). The two approaches were conducted independently.

2 All values were calculated using numerical simulation given 10 million samples of *A*, *B*, and *D* probabilities and values.

## A The Effect of Probability Weighting on Preference Reversals

Figure A1 shows that the probability weighting parameter α in Equation 4 has little effect on the preference reversal rate irrespective of the level of calculation noise. Preference reversals are observed for all plotted levels of α. In other words, preference reversals are predicted by an expected value maximizer irrespective of the presence or absence of bias in its estimate of its own subjective expected utility.[Fig-anchor fig14]

## B The Effect of RF Decoy Distance on Preference Reversals

Figure B1 shows that the preference reversal effect diminishes as a function of the distance of the RF decoy from the target choice. This is consistent with the effect observed by Soltani et al. (2012). All noise parameters were set to the values used in Attraction Effects.[Fig-anchor fig15]

## C The Effect of the Value Distribution Parameters on the Compromise Effect

Figure C1 shows the effect of the location and scale of the value distribution on the magnitude of the compromise effect. The compromise effect is smaller at higher values of location. The effect of location interacts with the effect of scale.[Fig-anchor fig16]

## D The Effect of the Compromise Decoy Distance on Preference Reversals

Figure D1 shows the effect of the distance of the compromise decoy from the target choice. Noise parameters were the fitted parameters derived in Attraction Effects. At distance = 0, the decoy is in a similarity decoy position and the preference reversal effect is, therefore, negative. The effect size increases as the decoy is moved away from the target choice and then plateaus.[Fig-anchor fig17]
